# Therapeutic Effects of Breviscapine in Cardiovascular Diseases: A Review

**DOI:** 10.3389/fphar.2017.00289

**Published:** 2017-05-23

**Authors:** Jialiang Gao, Guang Chen, Haoqiang He, Chao Liu, Xingjiang Xiong, Jun Li, Jie Wang

**Affiliations:** ^1^Department of Cardiology, Guang’anmen Hospital, China Academy of Chinese Medical SciencesBeijing, China; ^2^Graduate School, Beijing University of Chinese MedicineBeijing, China

**Keywords:** breviscapine, Chinese medicine, ethnopharmacology, herbal medicine, herbal active compounds

## Abstract

Breviscapine is a crude extract of several flavonoids of *Erigeron breviscapus (Vant.) Hand.-Mazz.*, containing more than 85% of scutellarin, which has been traditionally used in China as an activating blood circulation medicine to improve cerebral blood supply. Accumulating evidence from various *in vivo* and *in vitro* studies has shown that breviscapine exerts a broad range of cardiovascular pharmacological effects, including vasodilation, protection against ischaemia/reperfusion (I/R), anti-inflammation, anticoagulation, antithrombosis, endothelial protection, myocardial protection, reduction of smooth muscle cell migration and proliferation, anticardiac remodeling, antiarrhythmia, blood lipid reduction, and improvement of erectile dysfunction. In addition, several clinical studies have reported that breviscapine could be used in conjunction with Western medicine for cardiovascular diseases (CVDs) including coronary heart disease, myocardial infarction, hypertension, atrial fibrillation, hyperlipidaemia, viral myocarditis, chronic heart failure, and pulmonary heart disease. However, the protective effects of breviscapine on CVDs based on experimental studies along with its underlying mechanisms have not been reviewed systematically. This paper reviewed the underlying pharmacological mechanisms in the cardioprotective effects of breviscapine and elucidated its clinical applications.

## Introduction

**FIGURE 1:**
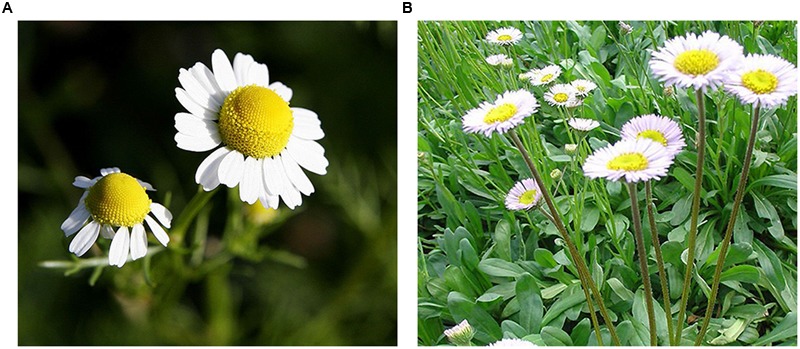
**Erigeron breviscapus (A)** flower; **(B)** whole plant.

*Erigeron breviscapus* (*Erigeron breviscapus (Vant.) Hand.-Mazz.*), also known as *Herba Erigerontis* or *Lamp Chrysanthemum*, is a traditional Chinese herb that has been in use for more than 600 years, found in Yunnan, Sichuan, Guizhou, and other southwest provinces of China. It belongs to the daisy family, which is a perennial, clump-forming herb that can grow up to 50 cm (20 inches) tall, though in some cases it can be less than 1 cm (0.4 inches) tall. In addition, its flower heads have blue, purple, or white ray florets surrounding yellow disk florets (as shown in **Figure 1**). The dried whole plant of *Erigeron breviscapus* has been used in folk medicine for the treatment of paralysis, rheumatism, gastritis, toothache, and fever (Yunnan Institute of Materia Medica, 1976).

Breviscapine is a crude extract of several flavonoids of *Erigeron breviscapus (Vant.) Hand.-Mazz.* (Zhang et al., 1988) that can be prepared into various forms including injection, granules, ordinary tablets, dispersible tablets, capsules, mixture, drop pills (Tian et al., 2014). To the best of our knowledge, the main active ingredient of breviscapine is scutellarin (Zhang et al., 1988). The use of breviscapine for the treatment of hypertension, cerebral embolism, and paralysis due to cerebrovascular accident dates back to the 1970s (Yunnan Institute of Materia Medica, 1976). Recent studies have suggested that breviscapine can be used to treat cerebral infarction and diabetic nephropathy. A meta-analysis of randomized and quasi-randomized controlled trials compared breviscapine plus routine therapy with routine therapy alone and showed a statistically significant benefit of using breviscapine for patient outcomes, with a marked neurologic improvement (Yang et al., 2012). Meanwhile, another meta-analysis of therapy combining breviscapine with mecobalamin for diabetic peripheral neuropathy suggested that the therapeutic efficacy of the combination was superior to mecobalamin alone (Liu et al., 2016). Another meta-analysis of the effect of breviscapine injection on the clinical parameters of diabetic nephropathy (Zheng et al., 2015) found significant renal protective effects (reduction in urine protein, serum creatinine and blood urea nitrogen) and adjustment for dyslipidaemia (effect on the levels of cholesterol, triglycerides (TG), and high-density lipoproteins).

Currently, because of its cardiovascular pharmacological effects (**Tables 1, 2**) and clinical benefits (**Table 3**), breviscapine has been extensively used in conjunction with Western medicine for the treatment of ischaemic cardiovascular disorders, such as angina pectoris and myocardial infarction (MI), in China (Cao et al., 2008; He et al., 2012). A meta-analysis to evaluate the efficacy and safety of breviscapine as an adjuvant therapy for patients with angina pectoris suggested that compared with the control group, the treatment group was superior in benefiting the patients with angina pectoris (Nie et al., 2012). In addition, breviscapine has been reported to have a broad range of cardiovascular pharmacological effects, including vasodilation, anti-thrombotic action, and platelet aggregation, anti-coagulation, scavenging of free radicals, and improvement in microcirculation, through various *in vivo* and *in vitro* experiments. Breviscapine has a series of pharmacological properties and is a kind of mixture of several flavonoids that can be used in clinical practice, but its underlying mechanism is still unclear.

**Table 1:** *In vivo* cardiovascular effects of breviscapine and scutellarin.

Effects	Compounds	Animal/Organs	Target	Reference
Protective effect against I/R	Scutellarin/ breviscapine	Male Sprague–Dawley (SD) rats	Myocardial infarction (MI) size myocardium cell apoptosis;	Lin et al., 2007
Protective effect against I/R	Breviscapine	I/R injury rats	PI3K/Akt/eNOS signaling pathway.	Wang J. et al., 2015
Protective effect against I/R	Breviscapine	Left heart I/R rats	IL-18 and ICAM-1	Wang Y. et al., 2013
Anti-inflammatory effect	Breviscapine	Myocardial I/R in New Zealand rabbits	Protein TNF-α and NF-κB	Zhao, 2010
Anti-inflammatory effect	Breviscapine	I/R rats	Protein TNF-α and IL-6	Gong et al., 2013
Anticoagulation	Breviscapine	Mice	Coagulation time (CT); prothrombin time (PT); platelet factor III (PF3); euglobulin lysis time (ELT)	Wang et al., 2003
Antithrombotic effect	*Erigeron breviscapus* flavones	Rats/rabbits	ADP, AA, and platelet activating factor (PAF)	Shen et al., 2000
Antithrombotic effect	Scutellarein	Rats	ADP-induced platelet	Song et al., 2011
Endothelial protective Effect	Dengzhan Xixin injection	Wistar rats	TNF-α; inflammatory reaction	Zhang et al., 2009
Myocardial protective effect	Breviscapine	Pressure-overload-induced cardiac Hypertrophy in mice	PKC-alpha-dependent ERK1/2 PI3K/AKT signaling	Yan et al., 2010
Myocardial protective effect	Scutellarin	Rats	Cardiac endothelial-mesenchymal transition Notch pathway	Zhou et al., 2014
Myocardial protective effect	Breviscapine	Streptozotocin-induced diabetic rats	Protein kinase C (PKC); phospholamban (PLB); protein phosphatase inhibitor-1 (PPI-1); Ca(2+)-ATPase (SERCA-2); ryanodine receptor (RyR)	Wang et al., 2010
Anticardiac remodeling effect	Breviscapine	Heart failure rats	Myocardial systolic and diastolic function	Li, 2011
Lipid-lowering Effect	Breviscapine	Diabetic rats	Blood lipids	Wei et al., 2010
Lipid-lowering effect	Breviscapine	Rabbits	The progress of intimal hyperplasia and atherosclerosis	Lou and Liu, 2009
Improving erectile function	Breviscapine	Spontaneously Hypertensive rats (SHR)	RhoA/Rho-kinase pathway	Li et al., 2014

**Table 2:** *In vitro* cardiovascular effects of breviscapine and scutellarin.

Effects	Compounds	Cells/tissues	Target	Reference
Vasodilating effect	Breviscapine	Rat aortic smooth muscle cells (ASMCs)	Ca2+-dependent K+ channel Channel open probability (Po) channel conductance	Xiuqin, 2006
Protective e effect against I/R	Breviscapine	Serum and myocardial tissues	ICAM-I protein in myocardium Na(+)-K(+)-ATPase, Mg(2+)-ATPase, Ca(2+)-ATPase in myocardial mitochondria	Jia et al., 2008
Anticoagulation	Breviscapine	Endothelial cells	Thrombomodulin	Zhou et al., 1992
Endothelial protective effect	Breviscapine	Human umbilical vein endothelial cells	Antioxidant effects; NF-κB activation	Chen et al., 2015
Endothelial protective effect	Scutellarin	Human umbilical vein endothelial cells	increase of VEGF	Lin et al., 2011
Myocardial protective effect	Breviscapine	Cardiomyocytes subjected to hypoxia	LDH leakage Intracellular free Ca2+ levels apoptosis necrosis	Li et al., 2004
Myocardial protective effect	Breviscapine	Cultured neonatal rat cardiac myocytes	PKC-alpha-dependent ERK1/2; PI3K/AKT signaling	Yan et al., 2010
Reduction of smooth muscle cell migration and proliferation	Breviscapine	Rat aortic smooth muscle cells	Thrombin/thrombin receptor gene	Hou et al., 2009
Reduction of smooth muscle cell migration and proliferation	Breviscapine	Rabbit vascular smooth muscle cell (VSMC)	NF-κB activity of VSMC	Pang et al., 2004
Reduction of smooth muscle cell migration and proliferation	Breviscapine	VSMC	ERK1/2 MAPK signaling	He et al., 2012
Antiarrhythmic effect	Breviscapine	Rat ventricular myocytes	Potassium current (Ito)	Deng et al., 2008
Antiarrhythmic effect	Breviscapine	Rat ventricular myocytes	INa channel current	Tang et al., 2009
Vasodilating effect	Breviscapine	Isolated thoracic aortic ring of rat	Receptor-operated Calcium channel	Zheng et al., 1998
Antiarrhythmic effect	Breviscapine	Hypertrophic rabbit hearts	Transmural repolarization dispersion; (TDR) early after depolarization; (EAD) Torsades de pointes; (Tdp)	Bo et al., 2011

**Table 3:** Included trials of breviscapine for cardiovascular diseases.

Target^a^	Design^b^	Duration	Dose	Case/control	Primary outcome measures^c^	Reference
SAP	RCT	14 days	40 mg, qd	25/25	Typical symptoms, the improvement of ST-T in ECG and time of ST-T in dynamic electrocardiogram	Zhang and Zhang, 2012
UAP	RCT	2 weeks	20 ml, qd	53/51	The dosage of isosorbide dinitrate, ECG curative effect, WBHV, PV, FIB, hs-CRP, erythrocyte aggregation index	Shen et al., 2014
AMI	CCT	10 days	60 mg, qd	25/20	LVEF, peripheral vascular resistance and incidence rate of post-angina pectoris	Gu T.B. et al., 2002
AMI	RCT	14 days	100 mg, qd	60/60	The improvement of cardiac function, the incidence of cardiac adverse events	Yang and Chen, 2013
AMI	RCT	14 days	50 mg, qd	54/54	The time of exercise-induced electrocardiographic ST-segment depression, shorten of the duration of ST-segment depression	Wang et al., 2009
EH	RCT	4–6 weeks	40 ml, qd	25/25	Amount of the urinary NAG and β_2_-MG, Blood pressure	Wang, 2000
AHCH	RCT	14 days	10 ml, qd	39/39	Hematoma volume, edema area, scandinavian stroke scale (SSS)	Shi and Ding, 2009
AF	Case series	2 weeks	36 mg, qd	20/-	Heart rate	Han, 1999
Hyperlipidemia	Case series	2 weeks	25 mg, qd	25/-	TC, LDL-c, HDL-c and TG	Yu, 2011
Hyperlipidemia	Case series	4 weeks	30 ml, qd	36/-	TC, LDL-c, HDL-c and TG	Wen and Ruan, 2004
UPA and hyperlipidemia	RCT	2 weeks	50 mg, qd	30/32	Serum lipid, WBV and PV, the times of angina	Peng and Ye, 2011
Viral myocarditis	CCT	2 weeks	10 mg, qd	40/30	DC,CK-MB	Gu et al., 2014
Viral myocarditis	RCT	2 weeks	10 mg, qd	30/30	TNF-α	Wang and Wang, 2009
HF-NEF	RCT	10 days	40 mg, qd	50/50	BNP, LVEF, LVEDV, typical symptoms	Zhang F., 2014
HF	RCT	14 days	50 mg, qd	64/62	LVEF, 6-MWT	Tian, 2010
Severe heart failure	CCT	14 days	50 mg, qd	46/23	LVEF, LVEDV, 6-MWT	Li, 2007
PHD	RCT	28 days	40 mg, qd	42/41	bFGF, PaO_2_, mPAP	Gao and Liang, 2009
Decompensable chronic PHD	CCT	20 days	50 mg, qd	38/46	The ability of erythrocyte deformability and leukocyte activation	Kong et al., 2006
Acute exacerbation of PHD	CCT	2 weeks	20 mg, qd	104/104	WBV, FIB, typical symptoms	Cao et al., 2006

*^a^SAP, stable angina pectoris; UAP, unstable angina pectoris; AMI, Acute myocardial infarction; EH, Essential hypertension; AHCH, acute hypertensive cerebral hemorrhage; AF, Atrial fibrillation; HF, heart failure; HF-NEF, heart failure with normal ejection fraction; PHD, pulmonary heart disease. ^b^RCT, randomized controlled trial; CCT, clinical controlled trial. ^c^WBV, whole blood viscosity; PV, plasma viscosity; FIB, fibrinogen; WBHV, whole blood high viscosity; LVEF, left ventricular ejection fraction; TC, total cholesterol; TG, triglycerides; DC, deceleration capacity; LVEDV, left ventricular end-diastolic volume; 6-MWT, 6 min walking test; bFGF, basic fibroblast growth factor; PaO_*2*_, partial pressure of oxygen; mPAP, mean pulmonary artery pressure.*

## Methodology

The PubMed and SinoMed database were searched with the terms “Breviscapine” or “*Erigeron breviscapus*” or “*Herba Erigerontis*” or “*Lamp Chrysanthemum*” or “scutellarin” or “apigenin-7-*O*-glucuronide” or “dengzhanxixin” as “Title/Abstract” or the MeSH terms “Breviscapine” or “scutellarin-7-*O*-glucuronide”. Articles related to therapeutic effects in cardiovascular diseases (CVDs) were picked out manually. All articles with abstract were included and we applied no language restrictions.

## Chemical Constituents

**FIGURE 2:**
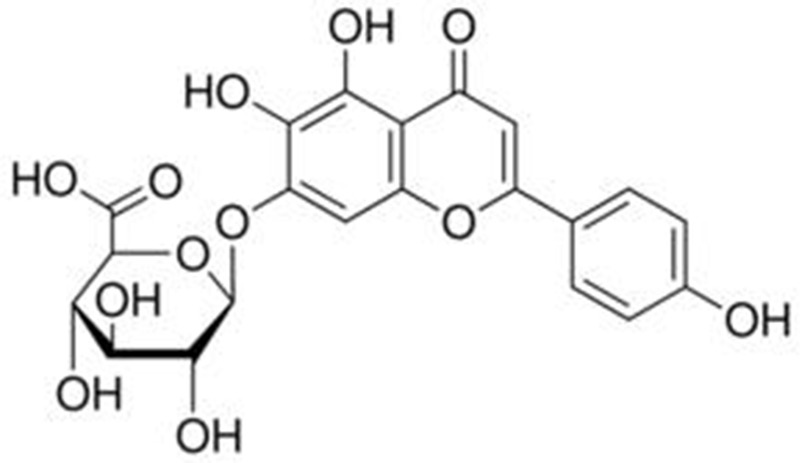
**Structure of scutellarin**.

**FIGURE 3:**
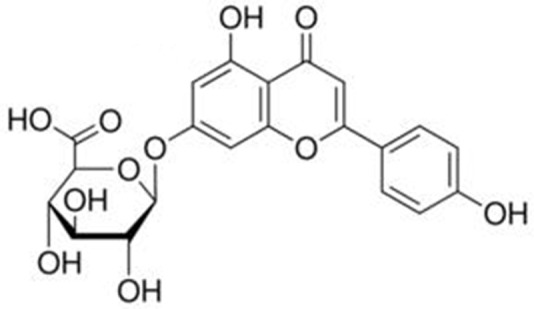
**Structure of apigenin-7-*O*-glucuronide**.

Breviscapine mainly contains scutellarin (4′,5,6,7-tetrahydroxyflavone-7-*O*-glucuronide) and apigenin-7-*O*-glucuronide. Scutellarin is the primary active ingredient. Its molecular formula is C_21_H_18_O_12_, and its relative molecular mass is 462.35. Its chemical structure is shown in **Figure 2**. However, scutellarin has low aqueous solubility, poor chemical stability, short biological half-life and rapid elimination rate from the plasma (Hao et al., 2005; Lu et al., 2010). The chemical structure of apigenin-7-*O*-glucuronide is shown in **Figure 3**; its molecular formula is C_21_H_18_O_11_ and the relative molecular mass is 446 (Wu, 2011).

## Cardiovascular Effects

### Vasodilating Effect

Vasculatures performing the function of maintaining vascular homeostasis play a vital role in both maintaining the blood pressure and providing the appropriate haemoperfusion according to dynamic physical conditions. As one of its regulatory mechanisms, the relaxation of vascular smooth muscles (VSM) can be triggered by the release of a series of endothelium-dependent and non-endothelium-dependent factors and has been demonstrated to be related to α-receptor, β-receptor, Ca^2+^ channel, and Ca^2+^-dependent K^+^ channel on the cell membrane (Furchgott, 1983; Rapoport et al., 1983; Tare et al., 1990; Bolotina et al., 1994). Based on *in vitro* studies, it has been concluded that breviscapine can relax norepinephrine-induced vasoconstriction in a concentration-dependent manner without influencing the function of the endothelium and without adjusting the α-receptors and β-receptors, even though it has been suggested that its vasodilation effect might be associated with the inhibition of the receptor-operated calcium channel (Zheng et al., 1998). There is another *in vivo* study showing that calcium activated potassium channels (K_Ca_) can be activated by the application of breviscapine in rat aortic smooth muscle cells (ASMCs) via promoting the open probability (Po) of the channel and enhancing channel conductance (Xiuqin, 2006).

### Protective Effect against Ischaemia/Reperfusion (I/R)

I/R injury often manifests as an aggravated endothelial impairment, leading to accelerated cardiomyocyte apoptosis or death, which can be measured by the size of MI (Kong et al., 2016; Yu et al., 2016). Studies have demonstrated that the protective effects of scutellarin alone on cardiovascular ischaemia are better than breviscapine with regards to the size of MI and myocardium cell apoptosis in MI rats, and its effects are dependent on the dose (Lin et al., 2007). The development of myocardial I/R injury has been shown to involve multiple mechanisms, including interference of specific pathways regulating the expression of some genes and activating relevant ATPase. One study suggested that breviscapine could provide significant protective effect against MI I/R injury, with the mechanism potentially involving the suppression of apoptosis of cardiomyocytes through the activation of the PI3K/Akt/eNOS signaling pathway (Wang J. et al., 2015). Moreover, as suggested by another study, breviscapine could inhibit the expression of IL-18 and ICAM-1 in protecting the lungs from inflammatory cascades (Wang Y. et al., 2013). In addition, the protective effects of breviscapine has been closely linked to the scavenging of oxygen free radicals, decreasing the expressions of ICAM-I protein in the myocardium and increasing the activities of Na(+)-K(+)-ATPase, Mg(2+)-ATPase, Ca(2+)-ATPase in the myocardial mitochondria (Jia et al., 2008).

### Anti-inflammatory Effect

Inflammatory processes play an important role in the development of CVDs and their associated complications (Ruparelia et al., 2017). Atherosclerosis is considered as an inflammatory disease (Ross, 1999). Many biological factors such as inflammatory cytokines, enzymes, and other mediators have been shown to be related to the effects of atherosclerosis (Walsh, 2003). It has been demonstrated that breviscapine is able to treat coronary disease and reduce the associated inflammatory response. The observed anti-inflammatory effects of breviscapine were demonstrated by a study comparing ischemic preconditioning with breviscapine and ischemic preconditioning alone; the combination treatment had better effect on decreasing the expression of TNF-α and NF-κB and reducing the injury due to inflammation to achieve myocardial protection during myocardial I/R in New Zealand rabbits (Zhao, 2010). Similarly, it could also decrease the expression of TNF-α and IL-6 to reduce the injury of I/R in rats (Gong et al., 2013).

### Anticoagulation Effect

The coagulation system and the anticoagulation and fibrinolytic systems interact dynamically, playing a major role in physiological haemostasis. On the other hand, this interaction might also be a common thread in a wide range of diseases, i.e., it might contribute to the pathology of various diseases, especially heart disease, cancer and inflammation (Marx, 1982). There is evidence that breviscapine can simulate fibrinolysis and anticoagulation of endothelial cells as indicated by the induction of thrombomodulin (TM) production and the downregulation of the expression of TM on the surface of the cells as well as the inhibition of TM release from the cells (Zhou et al., 1992). In addition, another study showed that the breviscapine extract influenced anticoagulation by significantly delaying the coagulation time (CT) and prothrombin time (PT), inhibiting the activity of platelet factor III (PF3) and decreasing the euglobulin lysis time (ELT); additionally, it could enhance the activity of fibrinolysis (Wang et al., 2003).

### Antithrombotic Effect

Pathogenic thrombi are responsible for acute clinical atherothrombotic diseases, such as acute coronary syndromes and ischaemic stroke. The activated platelets play a crucial role in the formation of pathogenic thrombi. During the process of platelet activation, specific agonists including thromboxane A2 (TxA2), adenosine diphosphate (ADP) and thrombin are associated with their corresponding receptors on the surface of platelets. Patients who suffered from atherothrombosis benefitted from the use of oral antiplatelet agents targeting the TxA2 (aspirin) and ADP (P2Y12 inhibitors such as clopidogrel, ticlopidine) platelet activation pathways (Fintel, 2012). One research showed that the *Erigeron breviscapus* flavones could significantly inhibit ADP, arachidonic acid (AA) and platelet activating factor (PAF) from forming into a thrombus (Shen et al., 2000). Meanwhile, another study suggested that scutellarin could prevent thrombosis and platelet aggregation and improve the characteristics of haemorheology by restricting the ADP-induced platelet aggregation rate in rats in a dose-dependent manner (Song et al., 2011).

### Endothelial Protective Effect

Vascular endothelial cells (VECs) are important for the endocrine system and target organs (Salles et al., 2016). Damage to VECs can cause various vascular dysfunctions, frequently accompanied by endothelial cell injury, production of oxygen free radicals and release of inflammatory cytokines. One study showed that Dengzhan Xixin injection (the main ingredient is breviscapine) could reduce the damage of TNF-α to cardiac micro VECs by inhibiting the inflammatory reaction (Zhang et al., 2009). Meanwhile, breviscapine was shown to have a protective role in ox-LDL-induced endothelial cell injury, which may be related to its antioxidant effects and inhibition of NF-κB activation (Chen et al., 2015). Vascular Endothelial Growth Factors (VEGFs), the most potent angiogenic factors in both physiological and pathological angiogenesis, play a crucial step in the process of repair after injuries. Similarly, another study suggested that scutellarin had a protective effect on VECs after ischaemia-reperfusion injury, and the mechanism may be related to the early increase of VEGF (Lin et al., 2011).

### Myocardial Protective Effect

When myocardial cells become injured by pathological factors such as cardiac surgery, ischaemia-reperfusion injury, diabetic injury, hypoxia injury, the pathological process may evolve from the initial cellular edema, degeneration, and necrosis into cardiac hypertrophy and myocardial fibrosis. There are several studies elucidating the potential myocardial protective effect of breviscapine and its mechanism. One study indicated that breviscapine favored myocardial protection by significantly reducing the LDH leakage, intracellular free Ca^2+^ levels, apoptosis and necrosis in cardiomyocytes subjected to hypoxia (Li et al., 2004). In case of diabetic injury, breviscapine may have a protective effect on diabetic cardiomyopathy by decreasing the expression of protein kinase C (PKC) and phospholamban (PLB), as well as increasing the expression of protein phosphatase inhibitor-1 (PPI-1), Ca(2+)-ATPase (SERCA-2), and ryanodine receptor (RyR) (Wang et al., 2010). In conditions of cardiac hypertrophy induced by angiotensin II (Ang II), it was demonstrated that breviscapine may still have a protective potential against cardiac hypertrophy by disrupting PKC-alpha-dependent ERK1/2 and PI3K/AKT signaling both in cardiac myocytes *in vitro* and mice *in vivo* (Yan et al., 2010). Another study also suggested that scutellarin could prevent isoprenaline-induced myocardial fibrosis by the inhibition of cardiac endothelial-mesenchymal transition, which may be associated with the Notch pathway (Zhou et al., 2014).

### Reduction of Smooth Muscle Cell Migration and Proliferation

Vascular smooth muscle cell (VSMC) migration and proliferation is a major pathophysiological step in the development of atherosclerosis. In addition, modulation VSMC proliferation might have therapeutic effects for vascular diseases (Ross, 1993). Thrombin has been shown to induce VSMC proliferation. Thrombin receptor is present in all the cell types that respond to thrombin, including platelets, endothelial cells and VSMC. In addition, thrombin receptor antagonists could be used as therapeutic agents that might have value by specific inhibition of cellular proliferation (Pakala et al., 2001). There was a research suggesting that breviscapine could significantly inhibit the proliferation of rat aortic smooth muscle cells to induce thrombin. And the possible mechanism is blocking the expression of thrombin receptor gene (Hou et al., 2009). In addition, breviscapine could obviously inhibit the proliferation of VSMC and may prevent atherosclerosis, and the mechanism may be realized partly by regulating NF-κB activity of VSMC (Pang et al., 2004). Furthermore, breviscapine could ameliorate high glucose-induced proliferation and migration of VSMCs via inhibiting ERK1/2 MAPK signaling (He et al., 2012).

### Anticardiac Remodeling Effect

Ventricular remodeling is the process of pathological repair, ventricular compensation and a secondary pathophysiological response that is accompanied by a series of ventricular myocardial injury-associated changes in parameters including size, shape, wall thickness and tissue structure. One study suggested that breviscapine could regulate ventricular remodeling in heart failure animal by improving the myocardial systolic and diastolic function (Li, 2011).

### Antiarrhythmic Effect

Breviscapine has been shown to advance certain kinds of specific antiarrhythmic effects on the rabbit heart and rat ventricular myocytes, even though the underlying mechanism behind the effects remains unclear, and is still under research. One study showed that breviscapine could diminish the transmural repolarization dispersion (TDR) and reduced the incidence of early after depolarization (EAD) and torsades de pointes (Tdp), which decreased the incidence of ventricular arrhythmias in hypertrophic rabbit hearts (Bo et al., 2011). It has been demonstrated that cardiac electrical activities depend on the ion channels on membranes of cardiac cells for their physiological function. Then, concerns have been raised about affecting the potassium and sodium currents in ventricular myocytes. It has been observed that breviscapine could inhibit potassium current (I_to_) in a concentration- and voltage-dependent manner (Deng et al., 2008) and I_Na_ channel current in a concentration-dependent manner (Tang et al., 2009), which might be an important mechanism in its antiarrhythmic effect.

### Lipid-Lowering Effect

Lipid-lowering is a routine treatment in CVDs. Some clinical studies have shown that breviscapine can reduce blood lipids. However, there have been different results in animal experiments. One study has shown that breviscapine could reduce blood lipid levels in diabetic rats (Wei et al., 2010). Another study suggested that it could inhibit the progress of intimal hyperplasia and atherosclerosis but could not reduce serum cholesterol levels (Lou and Liu, 2009). Additionally, there is no research explaining its mechanism of its lipid-lowering effect.

### Improving Erectile Dysfunction

Impaired erectile response is one of the potential complications of essential hypertension. As mentioned in the 2013 ESH/ESC guidelines for the management of arterial hypertension (ESH/ESC Task Force for the Management of Arterial Hypertension, 2013), erectile dysfunction can be considered as an independent cardiovascular risk factor and an early diagnostic indicator for clinical organ damage. Therefore, efforts have been focused on finding out whether breviscapine could reverse hypertension-induced erectile dysfunction. It has been concluded that the impaired erectile response in spontaneously hypertensive rats (SHR) may be caused by the increased signaling by RhoA/Rho-kinase and decreased signaling by nitric oxide (NO). One study showed that breviscapine could improve erectile function by downregulating the RhoA/Rho-kinase pathway (Li et al., 2014).

## Breviscapine for the Treatment of Clinical CVDs

Cardiovascular diseases are still the leading cause of death worldwide. Of the 57 million global deaths in 2008, more than 17.3 million (30%) were due to CVDs. Although the cardiovascular mortality rate has declined in many high-income countries in the past 2 decades, it has rapidly increased in low- and middle-income countries due to the lack of population-wide primary prevention and individual healthcare intervention (Mendis et al., 2011). Breviscapine is widely used in CVD prevention in China because of its effects on vasodilation, myocardial protection, anti-arrhythmia, decreasing arterial blood pressure, etc. Numerous studies have provided evidence to support these favorable effects; however, several studies have also reported adverse reactions, such as skin rashes, allergic shock, atrial fibrillation (AF), and diarrhea, occurring in CVDs (Liu and Bai, 2012; Zhang et al., 2016). This paper critically examines the scientific literature which reported the effects of breviscapine on cardiovascular diseases [coronary heart disease (CHD), MI, hypertension, arrhythmia, etc.]. Based on the methodology to perform this review, 19 trials were included in this review, among which there were 2 trials in CHD, 3 trials in MI, 2 trials in hypertension, 1 trial in arrhythmia, 3 trials in hyperlipidaemia, 2 trials in viral myocarditis (VMC), 3 trials in chronic heart failure (CHF), and 3 trials in pulmonary heart disease (PHD) (shown in **Table 2**).

### Coronary Heart Disease

Coronary heart disease is the most common type of CVD and one of the fatal diseases. The latest data showed that CHD led to 8.14 million deaths, which accounted for 16.8% of all deaths globally in 2013 (GBD 2013 Mortality and Causes of Death Collaborators, 2015). Currently, by changing lifestyle, such as exercising, having a healthy diet, treating hypertension, and medications, including anti-platelet drugs such as aspirin, nitro-glycerine, beta-blockers and statins, the morbidity of CHD has been reduced to some extent. However, these drugs also have some inevitable adverse effects. Breviscapine is a complementary medicine that has been used in combination with conventional medicine to prevent and treat CHD for decades in China. It provides many benefits; for example, it improves the therapeutic effectiveness compared to conventional treatment alone, and it helps to decrease the dosage of several drugs that may cause adverse effects (Wang C. et al., 2015). A large number of randomized, controlled trials have been carried out to explore the effects of breviscapine on CHD.

In one randomized controlled trial, 50 patients with stable angina pectoris were randomly allocated into two groups that received breviscapine (40 mg/250 ml 0.9% sodium chloride, iv drip, qd) combined with standard medication (*n* = 25) or standard medication alone (*n* = 25) for 14 days. The outcome showed that the symptoms of angina, the change of ST-T in ECG and the change time of ST-T in dynamic electrocardiogram improved more in the test group than in the control group. Additionally, the improvements in haemorheology, such as whole blood viscosity (WBV), plasma viscosity (PV), fibrinogen (FIB), and serum lipids in the test group were more remarkable than the control group (Zhang and Zhang, 2012). Similarly, another RCT was conducted to test the efficacy of breviscapine in patients with unstable angina pectoris, who were randomly assigned to receive 20 ml breviscapine daily in addition to conventional Western medicine (*n* = 53) or the conventional Western medicine alone (*n* = 51) for 2 weeks. The results demonstrated that the dosage of isosorbide dinitrate in the test group was lower than that in the control group and the curative effect on the ECG was better in the test group. In addition, the whole blood high viscosity (WBHV), PV, erythrocyte aggregation index, FIB and hs-CRP in the test group were also lower than that in the control group. Nevertheless, the researchers had not found a significant difference in the whole blood low shear viscosity and erythrocyte rigidity index between the two groups. In addition, this study reported 4 cases of nausea and 1 case of palpitation in the control group and 3 cases of nausea and 2 cases of abdominal distension in the test group (Shen et al., 2014).

### Myocardial Infarction (MI)

MI, also known as acute myocardial infarction (AMI), is a heart attack caused by the blockage of blood flow to the heart due to a thrombus of a ruptured atherosclerotic plaque (Mendis et al., 2011). A study reported that the rate of MI has decreased globally between 1990 and 2010 (GBD 2013 Mortality and Causes of Death Collaborators, 2015). Although the morbidity and mortality of MI have been controlled to a large extent with early and effective preventive measures and interventions, there are still some problems in secondary prevention and rehabilitation of patients with MI. Chinese medicine (CM) shows some advantages in these aspects, such as improving the quality of life (QOL) and decreasing the rate of adverse events (Xu-Feng et al., 2010; Duan et al., 2012; Zhang Y.H., 2014). Several clinical studies with breviscapine have reported its effects on MI. A controlled clinical trial was designed to observe the efficacy of breviscapine in AMI patients who received either breviscapine (60 mg/d) with routine treatments (*n* = 25) or the routine treatments alone (*n* = 20) for a period of 10 days. The outcomes of left ventricular ejection fraction (LVEF), peripheral vascular resistance and incidence rate of post-angina pectoris were significantly different in the patients in the combination group compared to the patients in control group (Gu T.B. et al., 2002). Similarly, another RCT was conducted on 60 patients after percutaneous coronary intervention (PCI) who were treated with conventional medicine and breviscapine injection. The results showed that the proportion of cardiac function class ≤NYHA functional class II in the test group (88.3%) was higher than that in the control group (61.7%). In addition, the incidence of cardiac adverse events (MI, arrhythmia, death) was lower in test group (6.7%) compared to the control group (21.7%) (Yang and Chen, 2013). Another RCT was carried out to observe the effects of breviscapine on exercise tolerance in patients with AMI who have received a successful intravenous thrombolytic treatment. Ninety-eight patients were randomly assigned to receive breviscapine with conventional treatment or the conventional treatment alone for 14 days. The results of the treadmill exercise test showed a significant prolongation of the time of exercise-induced electrocardiographic ST-segment depression (≥0.1 mV) and shortening of the duration of ST-segment depression in the combination group than the control group on the 36th day. However, there was no significant difference on the 14th day. This implied that breviscapine might have sustained effects (Wang et al., 2009).

### Hypertension

Hypertension results in 7.8 million deaths annually, accounting for 12.8% of the total deaths worldwide. The size of the population with uncontrolled blood pressure has grown from 6 million to nearly one billion between 1980 and 2008 (Mendis et al., 2011). Uncontrolled blood pressure is the major contributor not only to CHD and stroke but also to heart failure, chronic kidney disease, among others (Poulter et al., 2015). Herbal medicine combined with anti-hypertensive drugs are being increasingly used as an integrative therapy to control blood pressure and associated complications in both Eastern and Western countries (Ernst, 2005; Wang et al., 2012). A clinical trial studied the effects of erigeron injection on the renal function of elderly patients with essential hypertension. The result demonstrated that erigeron injection (the main ingredient is breviscapine) (40 ml, qd) had similar anti-hypertensive effect as enalapril (20 mg, qd). In addition, urinary NAG and β_2_-MG significantly decreased in the breviscapine group, which indicated that breviscapine might improve the tubular function of these patients (Wang, 2000). Another RCT aimed to investigate the efficacy of breviscapine in acute hypertensive cerebral hemorrhage patients. The patients were treated either with breviscapine plus routine Western medicine (*n* = 39) or routine Western medicine alone (*n* = 39) for 2 weeks. Outcomes of the haematoma volume, edema area and Scandinavian stroke scale (SSS) in breviscapine group were statistically better than those in control group (Shi and Ding, 2009).

### Arrhythmia

In the clinical practice, most of the therapies for arrhythmia are medically indicated. However, AF, a serious type of arrhythmia (Munger et al., 2014), is still not easy to address. AF is a major cause of sudden cardiac death, which accounts for half of the death due to CVDs worldwide (Mehra, 2007). Although the anti-arrhythmic effect of breviscapine has been investigated in several animal studies, there are a few clinical trials. One case series of 30 elderly patients with persistent AF who received erigeron injection (36 mg, iv drip, qd) for 2 weeks reported that the heart rate in the patients decreased from 115.4 ± 8.2 to 83.3 ± 7.6 after the treatment. The reported adverse events included three cases of dizziness, which were spontaneously resolved (Han, 1999).

### Hyperlipoidaemia

Hyperlipidaemia is abnormal increase of lipids in the blood, usually referred to the elevation of serum cholesterol and TG. High amount of cholesterol and TGs would increase the risk of CVDs. Data show that the prevalence of heart disease will decrease by 50% in 40-year-old men within five years if the serum cholesterol was reduced by 10% (Mendis et al., 2011). Thus, it is necessary to control the level of serum cholesterol and TGs. Animal studies have found that breviscapine can help lower elevated serum lipids. A clinical study was also carried out to observe its effects on patients with hyperlipidaemia. The results showed that the level of total cholesterol (TC), LDL-c, and TG decreased after a daily treatment with 25 mg breviscapine for 2 weeks. In contrast, the level of HDL-c increased (Yu, 2011). Another trial studied 36 elderly patients with hyperlipidaemia who received erigeron injection (30 mg, qd) for 2 weeks. A similar result was obtained (Wen and Ruan, 2004). Another RCT was designed to investigate the effect of breviscapine in patients with unstable angina pectoris with hyperlipidaemia. Neither the test group nor the control group received statins. The outcome of the serum lipid, WBV and PV showed statistically significant differences in the test group. Moreover, the duration of angina also decreased in the test group (Peng and Ye, 2011).

### Viral Myocarditis

Viral myocarditis is an inflammation in the cardiac muscle due to a viral infection. It contributes to the development of heart failure. Currently, symptomatic treatment is the major treatment of VMC and other therapies, such as intravenous immunoglobulin (IVIG) or herbal medicine have not shown any evidence-based benefits (Robinson et al., 2005; Liu et al., 2012). Several studies have demonstrated the effects of breviscapine on VMC. A randomized controlled trial was designed to study the effect of breviscapine injection on the deceleration capacity (DC, a technique to quantitatively detect autonomic nerve tension) of the heart rate in children with VMC with daily administration of 10 mg breviscapine (*n* = 30) or 100 U coenzyme A (CoA) and 40 mg adenosine triphosphate (ATP) (*n* = 30) for 2 week. The results showed that there was a significant elevation of the DC in the breviscapine group compared to the control group. The investigators also found a more marked decrease in CK-MB in the test group than in the control group (Gu et al., 2014). Another study reported similar results; the investigators demonstrated the TNF-α, a cytokine that can reflect the degree of inflammation in the myocardium (Lenzo et al., 2001), noticeably dropped in the breviscapine group (Wang and Wang, 2009).

### Chronic Heart Failure

Chronic heart failure often occurs at the terminal stage of most CVDs. Epidemiological survey shows that the prevalence of CHF in adults in the developed countries is approximately 2%, and in China, it is 0.9% (Gu et al., 2002; Mcmurray and Pfeffer, 2005). Although 30–40% of the patients die within a year of CVD diagnosis, the mortality is less than 10% annually. The key problem that needs to be solved is the impact on the QOL, such as mood disorder (National Clinical Guideline Centre, 2010). In a randomized controlled trial, 100 stage NYHA III∼IV patients with heart failure with normal ejection fraction (HF-NEF) were instructed to take 40 mg/d breviscapine plus routine medication or the routine medication alone for 10 days. The outcome parameters demonstrated that the B-type natriuretic peptide (BNP) decreased more in the test group. However, there was no difference in the LVEF and left ventricular end-diastolic volume (LVEDV) between the test and control groups. In addition, the typical symptoms such as shortness of breath, chest tightness, fatigue, and weakness improved a lot in the test group than in the control group (Zhang F., 2014). One RCT was conducted in 126 stage NYHA II∼III patients who were randomly given breviscapine (50 mg, qd) with conventional medicine or the conventional medicine alone for 2 weeks. The results showed that the LVEF and 6-minute walk test (6 MWT) in the combination group was markedly better compared to the control group (Tian, 2010). Another clinical trial studied 46 stage NYHA III∼IV patients with severe heart failure and obtained similar results (Li, 2007).

### Pulmonary Heart Disease

Pulmonary heart disease leads to heart failure and/or respiratory failure. The pressure afterload is an initial step of the disease (Voelkel et al., 2013). At present, antibiotics, oxygen therapy, anticoagulants and vasodilators are the major treatments for PHD. Several studies have indicated the safety and effectiveness of Chinese medicine (Shenmai injection) combined with conventional treatment in this disease (Shi et al., 2015). There have also been some studies reporting the effects of breviscapine on PHD. A randomized controlled trial investigated the effects of breviscapine on 83 patients with PHD who were treated with conventional medicine plus 40 mg/d breviscapine or the conventional medicine alone for 28 days. The results demonstrated that the basic fibroblast growth factor (bFGF, a polypeptide that can induce vascular endothelial growth factor), partial pressure of oxygen (PaO_2_) and the mean pulmonary artery pressure (mPAP) noticeably improved in the breviscapine group (Gao and Liang, 2009). Another trial also observed the effects of breviscapine on PHD patients; the outcome of the erythrocyte deformability and leukocyte activation showed a significant difference between the breviscapine group and the control group. It has been suggested that breviscapine may prevent the progression of PHD by improving the erythrocyte deformability and leukocyte activation, which can affect the serum hypercoagulation state of patients with decompensated chronic PHD (Kong et al., 2006). One clinical trial investigated the effects of breviscapine on patients with acute exacerbation of PHD. The results showed that symptoms such as dyspnoea, cough, edema and cyanosis improved more in the test group than in the control group. In addition, there was an obvious decrease in some of the indexes such as WBV and FIB in the test group, which demonstrated the improvement in blood viscosity (Cao et al., 2006).

## Dosage and Side Effects

Breviscapine is widely used in clinics in the form of injection and oral administration. The recommended dosages of injection range from 5 to 20 mg per day at one time, and the dosages of oral administration range from 120 to 240 mg per day divided into three times. Due to its poor water solubility and low bioavailability *in vivo*, many new delivery methods have been designed and developed, including dispersion tablet, drop pill, liposome, nanoparticle, nanoemulsion, and lipid emulsion (Zhong et al., 2005; Patel et al., 2012; Ma et al., 2015). The adverse reaction of breviscapine mostly occurs during injection. One meta-analysis of the adverse reactions of breviscapine included 33 clinical studies of 1761 patients. Overall, 72 adverse reactions were reported, with an incidence rate of 4.09%. The adverse reactions included allergies, skin itching, rash, facial flushing, chest tightness, palpitation, dizziness/vertigo, headache, and gastrointestinal complaints. However, in this study, the investigators also found that there were no significant differences on the adverse reactions of the breviscapine injection compared with the counterpart medications, especially within 15 days (Feng et al., 2016). With regard to drug interactions, breviscapine could inhibit phenacetin metabolism mediated by CYP1A2 during short-term *in vitro* experiments (Qin et al., 2012) and inhibit the activity of CYP3A4 *in vivo*. Breviscapine also significantly increased the plasma concentration of dapsone in rats (Liu et al., 2013). The clinical safety and reasonable application of breviscapine injection clearly states that breviscapine is incompatible with the following drugs: ampicillin sodium, gentamicin sulfate, chloramphenicol, ciprofloxacin lactate, magnesium sulfate, procaine hydrochloride, cefradine, low molecular weight dextran, furosemide, and acetic acid hydrogenated prednisone (Zhao et al., 2008).

## Conclusion and Perspective

Traditional Chinese medicines (TCMs) continue to play an important role in the prevention and treatment of cardiovascular diseases in China. Unlike Western medicine, the holistic, and synergistic nature of TCMs arise from their herbal components, which contain hundreds of compounds and exert their effects on diseases via the binding of multiple compounds to multiple different targets to improve their performance on the systemic intervention of complex diseases. However, the mechanism of TCMs remains unclear, which makes it difficult for the rest of the world to understand how they work and prevent their global applications. Therefore, studies at the level of herbs might be a good way to provide comprehensive understanding of TCMs. Previously, one of the main strategies to study a compound prescription in TCMs has been to study its mechanism. Due to the presence of multiple compounds, it is always unclear which of the ingredients are producing real effects. The monomer component of Chinese herbal medicine (CHM), also known as the natural pure compound drug, has recently attracted much attention. The natural extract artemisinin and its derivatives are good examples of monomer components of CHM that can treat diseases through various activities, and can be a good starting point to uncover the mechanism of TCMs.

Similarly, a large number of monomer components of CHM with cardiovascular actions have been studied over the last few decades. For example, several systematic reviews have been conducted pertaining to salvianolic acid B (Wang J. et al., 2013), tetramethylpyrazine (Ming et al., 2016), *Panax notoginseng* saponins (Yang et al., 2014), etc. Among them, scutellarin, the principal component of breviscapine, is a type of monomer component of CHM and breviscapine has significant effects on vasodilation (improving erectile function); protection against I/R; anticoagulation and antithrombosis; reduction of smooth muscle cell migration and proliferation; anticardiac remodeling; antiarrhythmia, and reduction of blood lipids. Breviscapine also has a protective effects on myocardial and endothelial structures because of its anti-inflammatory effects. In addition, by reviewing the clinical studies, we believe that the most remarkable feature of breviscapine is its ability to perform multiple functions in regulating blood vessels, which are associated with cardiovascular diseases, stroke, and diabetes.

Though breviscapine has a wide range of cardiovascular effects on the prevention and treatment of CVDs, there are also a few problems that we need to consider. First, the above experimental studies that we reviewed focused on one aspect of the mechanism of breviscapine and very few studies could draw a definitive conclusion due to the low methodological quality, and none of the studies validated the findings both *in vitro* and *in vivo*. There were more studies investigating its properties of vasodilation, I/R and anticoagulation and antithrombotic effect than the other mechanisms. However, the results on its lipid-lowering effect in animal experiments were different in two studies. In addition, there was no study investigating its relevant mechanism. Second, since the studies were mainly published in China, the strength of the evidence was limited by the lack of controls or placebos, non-randomization, non-blinded design, and/or small samples of patients. Therefore, multicentred, large samples, and randomized controlled trials need to be done to evaluate the efficacy and safety of breviscapine for CVDs. Third, the side effects of breviscapine mostly occurred when it was injected, suggesting that suitable forms of delivery should be considered. In addition, patients with acute cerebral hemorrhage or bleeding tendency were excluded. Overall, it is very important to investigate the use of breviscapine for the treatment of CVDs. Nevertheless, all these pressing problems should be addressed in future studies.

## Author Contributions

JG and GC designed the work of review; JG, GC, HH, and CL reviewed the literature available on this topic and wrote the paper; XX and JL contributed in the scientific writing of the manuscript; JG, GC, and JW revised the manuscript. All authors approved the paper for publication. JG, GC, HH, and CL contributed equally to this work.

## Conflict of Interest Statement

The authors declare that the research was conducted in the absence of any commercial or financial relationships that could be construed as a potential conflict of interest.
